# Perceptions of women and men in mixed-race heterosexual relationships

**DOI:** 10.1177/13684302241233505

**Published:** 2024-03-16

**Authors:** Maria Iankilevitch, Alison L. Chasteen

**Affiliations:** 1 University of Victoria, Canada; 2 University of Toronto, Canada

**Keywords:** couples, first impressions, interracial, mixed-race, relationships

## Abstract

Although the number of mixed-race couples is increasing in North America, these couples continue to experience stigma and discrimination, which can have deleterious effects on individuals in these relationships. In three samples, we examined perceivers’ first impressions of targets in mixed-race couples when viewed with their romantic partner versus alone, including their warmth and competence (Sample 1a), global morality (Sample 1b), and specific stereotypic behaviors including likelihood to betray, conform, and be prejudiced (Sample 1c). Partner effects occurred for specific stereotypes relevant for intergroup behaviors such that individuals in mixed-race couples were rated as more likely to betray close others and to be less conforming and less prejudiced than individuals in same-race couples when viewed with their partners. These results suggest that specific stereotypes relevant for intergroup relations are affected by the race of targets’ romantic partners and lay the foundation for understanding the unique challenges faced by members of mixed-race couples.

Although the number of mixed-race couples in the US is growing at an exponential rate (Barroso et al., 2020; Parker & Barroso, 2021), negative bias still exists against mixed-race couples (Skinner & Rae, 2019). Importantly, mixed-race couples experience unique forms of discrimination that are specific to the pairing of the two individuals. For instance, people evaluate mixed-race couples as less compatible (Lewandowski & Jackson, 2001), and mixed-race families as less family-like and less loving than same-race families (Kille & Tse, 2015). Furthermore, people exhibit responses of disgust when looking at images of mixed-race but not same-race couples (Skinner & Hudac, 2017). Such negative perceptions are consistent with news reports of mixed-race couples being threatened, physically attacked, and having their property vandalized (Leshan, 2020; MacDonald, 2010; Riess & Andone, 2019). Other couples have been refused wedding services (Zraick, 2019) and marriage licenses (U.S. Mixed Race Couple Denied, 2009), have been evicted from their homes (Mitchell, 2016), have had their homes appraised at an unfairly low rate (Scott et al., 2020), or have been banned from community groups entirely (Ng, 2011).

Though there is both anecdotal and scientific evidence demonstrating that prejudice persists against mixed-race couples, little is known about the relevant stereotypic first impressions associated with members of mixed-race couples. The first goal of this study was to examine how people explicitly rate members of mixed-race couples on general stereotypic perceptions as well as specific behavioral stereotypes. General stereotypic perceptions included perceptions of warmth (Sample 1a), competence (Sample 1a), and global morality (Sample 1b). Warmth and competence are well-established fundamental dimensions that systematically predict attitudes and behaviors towards individuals and groups, and have been very influential in person perception research (Cuddy et al., 2007; Fiske, 2018; Fiske et al., 2002). However, it has also been argued that people may be especially motivated to make morality-based judgements of targets because the “goodness” or “badness” of a target’s character may signal to perceivers whether the target may be helpful or harmful (e.g., Goodwin et al., 2014). Therefore, people may be quite sensitive to cues related to targets’ moral character (Ybarra et al., 2001). Indeed, a number of studies have found that morality information is more important in impression formation to perceivers than warmth and competence (Brambilla et al., 2011; De Bruin & van Lange, 2000; Goodwin et al., 2014; Hartley et al., 2016; Leach et al., 2007; Wojciszke et al., 1998; Ybarra et al., 2001). Taken together, the literature suggests that examining general perceptions of warmth, competence, and global morality is an important first step in understanding first impressions formed of others.

We also examined three specific stereotypic behavioral subfacets of morality: betrayal, conformity, and prejudice (Sample 1c). These specific subfacets were chosen because perceivers may make inferences about targets’ relations to their ingroups and outgroups based on the targets being in a same- or mixed-race relationship. For example, the very close positive ties that individuals in mixed-race relationships have with a member of a different racial group may lead to the perception of less ingroup loyalty or greater likelihood to betray one’s ingroup, as well as less prejudice towards outgroup members. Furthermore, mixed-race relationships are perceived as less traditional than same-race relationships (e.g., Lewandowski & Jackson, 2001), suggesting that perceivers may interpret that members of mixed-race couples are less inclined to follow social rules and traditions. Therefore, this study examined to what degree people believe that members of mixed-race (vs. same-race) couples would betray close others, would conform to societal traditions, and would be prejudiced.

Importantly, each member of a couple may be perceived differently and may consequently have different experiences. Therefore, the second goal of this study was to examine how people evaluate individual members in mixed-race relationships when presented alone versus with their romantic partners. To our knowledge, this is the first investigation of how first impressions of individuals in mixed-race relationships may differ depending on whether they are viewed with their partner versus alone. First impressions of novel others are formed very quickly and automatically (e.g., Freeman & Ambady, 2011), and these first impressions may have downstream consequences for subsequent attitudes and behaviors (e.g., Fiske & Neuberg, 1990). First impressions can be formed from a dynamic interaction between bottom-up processes (e.g., face cues from the target individual) and top-down processes (e.g., contextual information; Freeman & Ambady, 2011). For romantic couples, perceptions of a target individual may be affected by evaluations of the target themselves (i.e., bottom-up process) and evaluations of their romantic partner who provides contextual information (i.e., top-down process). For heterosexual mixed-race couples in particular, the Race x Gender of the target individual and the Race x Gender of their romantic partner may interact to predict first impressions of the target individual. Therefore, considering the intersectionality between members of mixed-race couples may be especially important for understanding first impressions of individuals in these relationships.

Intersectionality research involving diverse racial samples has demonstrated that overlapping or congruent stereotypes between gender and race lead to Black individuals being stereotypically perceived as most masculine, followed by White individuals, followed by East Asian individuals, who are stereotypically perceived as most feminine (Axt et al., 2022; Galinsky et al., 2013; Ghavami & Peplau, 2013; Johnson et al., 2012). This congruence between gender and race stereotypes may have implications for interracial dating between men and women. Galinsky et al. (2013) found that White heterosexual men are more attracted to femininity, while White heterosexual women are more attracted to masculinity, and that this may explain racial dating preferences. Indeed, both Auelua-Toomey and Roberts (2023) and Galinsky et al. (2013) found that White men are, on average, more attracted to Asian than Black women; and White women are, on average, more attracted to Black than Asian men as dating partners. Auelua-Toomey and Roberts (2023) further found that Asian and Black individuals were more attracted to potential White than to potential Black or Asian partners, respectively. Taken together, these findings point to two outcomes. One is that mixed-race couples involving one White partner would be most common. Indeed, demographic data show that about 80% of mixed-race couples in the US have one White partner (Livingston & Brown, 2017). Second, couples where gender and race stereotypes are congruent, such that the women have the more stereotypically feminine and the men have the more stereotypically masculine racial background, are more common than couples where gender and race stereotypes are not congruent. Indeed, demographic data from the U.S. Census and the Pew Research Center show that couples with White women–Black men and Asian women–White men are more common than couples with Black women–White men and White women–Asian men (Galinsky et al., 2013; Livingston & Brown, 2017). Based on the congruence between gender and race stereotypes, we made several hypotheses about perceptions of each partner.

## Present Study

Across three samples of participants, we examined first impressions of individuals in same-race versus stereotype-congruent mixed-race versus stereotype-incongruent mixed-race couples. We began by collecting photos of same-race Black, East Asian, and White couples as well as Black–White and East Asian–White mixed-race couples. We grouped the mixed-race couples into stereotype-congruent and stereotype-incongruent couples based on gendered racial stereotypes (Galinsky et al., 2013), such that White women–Black men and Asian women–White men were categorized as congruent mixed-race couples, and Black women–White men and White women–Asian men were categorized as incongruent mixed-race couples. Next, participants made first impression ratings of one member of each couple either about general stereotypes—which included perceived warmth (Sample 1a), competence (Sample 1a), and global morality (Sample 1b)—or about specific stereotypes of betrayal, conformity, and prejudice (Sample 1c). Some participants viewed the target individuals with their partners, and other participants viewed the target individuals alone.

Hypotheses for Sample 1a and Sample 1c were preregistered on Open Science Framework: https://osf.io/mchgy/ were preregistered on the Open Science Framework.^1^ We treated same-race couples as a control group, and thus predicted that perceptions of members of these couples on all dependent measures would be similar when presented with their partners versus alone. Below, we focus on hypotheses related to stereotype-congruent and stereotype-incongruent mixed-race couples and to the comparison between mixed-race and same-race couples (where applicable).

**Hypotheses for warmth (H1):** We predicted that warmth ratings would be linked to the perceived femininity of each individual relative to their partner. For congruent mixed-race couples, we predicted that women would be rated as warmer and men would be rated as colder when viewed with their partner versus alone, because the contrast between women with stereotypically more feminine racial backgrounds and men with stereotypically more masculine racial backgrounds would be more salient when these partners are viewed together. We predicted the opposite pattern of results for incongruent mixed-race couples, such that women would be rated as colder and men would be rated as warmer when viewed with their partner versus alone, because the stereotypically more masculine racial background of the women and the stereotypically more feminine racial background of the men would be more salient when partners are viewed together.
**Hypotheses for competence (H2), global morality (H3), betrayal (H4), and conformity (H5):** Recent research has shown that mixed-race couples are viewed less favorably than same-race couples by monoracial (but not multiracial) individuals (Skinner & Rae, 2019). Furthermore, mixed-race relationships are perceived as less family-like (Kille & Tse, 2015) and as less compatible (Lewandowski & Jackson, 2001) than same-race relationships, suggesting that individuals in mixed-race relationships may be viewed as choosing partners that match them poorly. Therefore, we predicted that individuals in same-race couples would be rated as more competent, more moral, less likely to betray close others, and more conforming to societal traditions than individuals in mixed-race couples when viewed with their partners. Furthermore, we predicted that individuals in mixed-race couples would be rated as less competent, less moral, more likely to betray close others, and less conforming when viewed with their partner versus alone. Finally, given that stereotype-incongruent mixed-race couples include individuals that are less stereotypically prototypical of their racial background (e.g., Schug et al., 2015) and are rarer than stereotype-congruent mixed-race couples (Galinsky et al., 2013; Livingston & Brown, 2017), we predicted that all these effects would be greater for incongruent than congruent mixed-race couples.
**Hypotheses for prejudice (H6):** One potential benefit of being in a mixed-race relationship is that of being viewed as less prejudiced. This is because engaging in an intimate relationship with a member of another racial background may be seen by perceivers as the target individual being open to having deep positive connections with members of their racial outgroups. Therefore, we predicted that individuals in mixed-race couples would be rated as less prejudiced when viewed with their partner versus alone, though we did not specify any predictions about individuals in stereotype-congruent versus stereotype-incongruent mixed-race couples. Furthermore, we hypothesized that individuals in mixed-race couples would be rated as less prejudiced than individuals in same-race couples when viewed with their partner.

## Methods

### Participants and Design

The study had a 3 (couple type: same-race, congruent mixed-race, incongruent mixed-race) × 2(target gender: woman, man) × 2(viewing condition: target alone, target with partner) mixed design with couple type being a repeated measures variable, and target gender and viewing condition being between-groups variables.

Participants were U.S. adults recruited via Prodege. We aimed to include at least 30 participants per between-groups condition (i.e., Target Gender × Viewing Condition), which would result in 30 ratings per target individual in each condition. This is because previous person perception research has shown that approximately 30 participants per target individual are needed for good interrater reliability (i.e., Cronbach’s alpha > .80; e.g., Tskhay & Rule, 2015), and for ratings of traits examined in this study to stabilize (Hehman et al., 2018). Furthermore, Judd et al. (2012) demonstrated that power increases very little in cross-classified studies when there are more than 30 participants per condition, which further confirms our goal of including 30 participants per target. We further wanted to gather data from White, East Asian, and Black participants; therefore, we aimed to analyze data from at least 360 participants. See Table 1 for summary of participants.

**Table 1.** Summary of participant demographics for each dependent variable in each sample.

	Sample 1a	Sample 1b	Sample 1c	Sample 1c
Dependent variable(s) per sample	Warmth competence	Global morality	Betrayal conformity	Prejudice
*N* (min. *N*)	463 (360)	402 (360)	402 (360)	413 (360)
White	155 (120)	134 (120)	134 (120)	136 (120)
East Asian	163 (120)	134 (120)	134 (120)	134 (120)
Black	145 (120)	134 (120)	134 (120)	143 (120)
*n* per b-g condition (min. *n*)				
White	30–45 (30)	33–34 (30)	33–34 (30)	33–36 (30)
East Asian	32–48 (30)	33–34 (30)	33–34 (30)	33–34 (30)
Black	30–40 (30)	33–34 (30)	33–34 (30)	33–42 (30)
Age				
Range	18–56	18–57	18–55	18–55
* M* (*SD*)	35.77 (10.12)	33.07 (8.89)	34.48 (9.05)	35.20 (9.38)
Gender				
Male	220	226	168	181
Female	240	174	233	232
Transgender	3	2	1	0
Total data points				
Warmth	64,799	-	-	-
Competence	64,781	-	-	-
Morality	-	56,254	-	-
Betrayal	-	-	56,226	-
Conformity	-	-	56,268	-
Prejudice	-	-	-	57,793

*Note*. In Sample 1c, due to a technical error for perceptions of prejudice, we had to replace participants in one of the between-groups conditions with a new batch of participants, therefore, sample sizes are different than for perceptions of betrayal and conformity. Min. = minimum; b-g = between-groups.

Informed consent was obtained by having participants read and sign the consent form prior to beginning the study. The study was in compliance with the university’s of Toronto’s ethics committee.

### Procedure and Materials

Participants were told that they would view a series of engagement photos of heterosexual couples, and they were asked to focus only on the man or the woman in all the photos. For participants who viewed target individuals alone, we told participants that the targets’ partners had been cropped out of the picture to help them focus only on the target individual.

Participants then viewed 140 photos one at a time in random order and responded to a question about the target for each photo. Photos included same-race (White–White, East Asian–East Asian, Black–Black), congruent mixed-race (East Asian woman–White man, White woman–Black man), and incongruent mixed-race (White woman–East Asian man, Black woman–White man) couples, which resulted in seven gender by race couple types. We used 20 photos per gender by race couple type, which is in line with Johnson et al. (2012, Study 1), who used 22 photos per condition to test explicit perceptions of gender and race stereotypes. Selected photos were taken from newspapers and consisted of couples in which both members were facing the camera and smiling and did not have any facial accessories (i.e., piercings or glasses). The individuals in the photos ranged from 24 to 43 years old (*M*_age_ = 30.43, *SD*_age_ = 3.13). Photo stimuli were cropped from the top of the head to the bottom of the chin and from ear to ear on the sides or to frame the face if hair covered the ears. All photos were then resized to a height of 150 pixels. No further changes were made to the stimuli, to keep them ecologically valid.

There were six dependent variables assessed across the three samples. The general stereotype ratings included warmth and competence (Sample 1a) and global morality (Sample 1b). The specific stereotype ratings included likelihood to betray close others, to conform to societal traditions, and to be prejudiced (Sample 1c). All responses were made on a slider scale ranging from 0 to 100, with higher scores indicating more of that attribute. For warmth, participants answered the question, “How warm do you think the [woman/man] in the photo?” (0 = *very cold*, 100 = *very warm*). For competence, participants answered the question, “How competent do you think the [woman/man] in the photo is?” (0 = *very incompetent*, 100 = *very competent*). For global morality, participants answered the question, “How moral is the [woman/man] in the photo?” (0 = *not at all moral*, 100 = *extremely moral*). For betrayal, participants answered the question, “How much do you think the [woman/man] in this photo is likely to betray family and friends?” (0 = *not at all*, 100 = *a great deal*). For conformity, participants answered the question, “How much do you think the [woman/man] in this photo conforms to the traditions of society?” (0 = *does not conform at all*, 100 = *conforms a great deal*). For prejudice, participants answered the question, “How prejudiced do you think the [woman/man] in this photo is?” (0 = *not at all prejudiced*, 100 = *extremely prejudiced*).

Participants viewed the 140 photos in blocks such that each dependent variable appeared in its own block. In samples where we included more than one dependent variable (i.e., 1a and 1c), blocks were presented in randomized order. After responding to the dependent measures, participants completed several demographic questions, and were debriefed and compensated.

## Results

### Analytic Plan

We modeled participants’ ratings of each dependent variable as a function of couple type (three groups: same-race, congruent mixed-race, incongruent mixed-race), target gender (two groups: woman, man), and viewing condition (two groups: target alone, target with partner) using cross-classified multilevel models and controlling for participants’ race.^2^ We included participants and target photos as nesting variables and modeled random slopes of couple type, target gender, and condition to achieve more precise estimates of the fixed effects (Aguinis et al., 2013). Not all models converged, therefore, we trimmed the random effects in line with Bates, Klieglb, et al. (2015) until convergence was achieved (see supplemental material for details).

All models were estimated with an unstructured covariance matrix using the “lmer” function in the “lme4” package (Bates, Mächler, et al., 2015) in R Version 3.5.2 (R Core Team, 2018). Tukey’s honestly significant difference (HSD) test was used to conduct post hoc analyses using the “pairs” function, and estimated marginal means and standard errors were computed using the “emmeans” function in the “emmeans” package (Lenth et al., 2020). *R*-squared was calculated as a measure of effect size using the “effect_size” function in the “effectsize” package (Ben-Shachar et al., 2020). To avoid giving meaning to sampling error, we considered effects with *p* < .05 and at least a small effect size of *R*^2^ ⩾ .02 as being significant and meaningful, therefore, effects that did not achieve at least a small effect size were treated as nonsignificant regardless of *p* value (Cohen, 1988). The data and analysis codes are available on the Open Science Framework.^3^

### General Stereotypes

The results for perceived general stereotypes—which included warmth, competence, and global morality—had very few significant effects and largely did not support the hypotheses (H1–H3). Therefore, the combination of a target individual’s race and their romantic partner’s race may not affect perceptions when making broad stereotypic evaluations. Given the lack of significant effects, we describe these results in the supplemental material, and discuss findings of specific stereotypic behavior below.

### Specific Stereotypes

Unlike for broader, general stereotypes, several effects emerged for specific stereotypes regarding perceived betrayal (H4), conformity (H5), and prejudice (H6). See Table 2 for omnibus statistics, and the supplemental material for numerical table summaries of post hoc statistical findings. Note that all significant post hoc results reported below are significant at *p* < .05.

**Table 2.** Omnibus statistics for perceived betrayal, conformity, and prejudice.

Effect	*F*(*df*)	*p*	*R* ^2^
Betrayal			
Participants’ race (control)	0.75(2, 396)	.473	< .01
Couple type	7.81(2, 220)	< .001	.07
Target gender	24.77(1, 396)	< .001	.06
Viewing condition	16.78(1, 396)	< .001	.04
Couple Type x Viewing Condition	5.26(2, 402)	.006	.03
Couple Type x Target Gender	1.79(2, 402)	.168	< .01
Target Gender x Viewing Condition	0.64(1, 396)	.425	< .01
Three-way interaction	1.02(2, 402)	.361	< .01
Conformity			
Participants’ race (control)	6.37(2, 396)	.002	.03
Couple type	170.30(2, 137)	< .001	.71
Target gender	2.05(1, 396)	.153	< .01
Viewing condition	3.82(1, 396)	.051	< .01
Couple Type x Viewing Condition	1156.62(2, 55721)	< .001	.04
Couple Type x Target Gender	21.90(2, 55721)	< .001	< .01
Target Gender x Viewing Condition	0.30(1, 396)	.585	< .01
Three-way interaction	35.37(2, 55721)	< .001	< .01
Prejudice			
Participants’ race (control)	0.49(2, 407)	.614	< .01
Couple type	6.02(2, 309)	.003	.04
Target gender	5.55(1, 407)	.019	.01
Viewing condition	18.49(1, 407)	< .001	.04
Couple Type x Viewing Condition	32.86(2, 417)	< .001	.14
Couple Type x Target Gender	0.30(2, 417)	.743	< .01
Target Gender x Viewing Condition	7.95(1, 407)	.005	.02
Three-way interaction	0.13(2, 417)	.880	< .01

*Note. F* = *F* ratio; *df* = degrees of freedom; *p* = probability value.

#### Betrayal

A main effect of couple type emerged. Tukey’s HSD determined that, as predicted, individuals in same-race couples (*M* = 27.69, *SE* = 0.99) were rated as significantly less likely to betray close others than individuals in congruent mixed-race couples (*M* = 30.03, *SE* = 1.01). Furthermore, individuals in same-race couples were rated as marginally less likely to betray close others than individuals in incongruent mixed-race couples (*M* = 29.23, *SE* = 1.03). However, there was no significant difference between congruent mixed-race and incongruent mixed-race couples on perceived likelihood to betray close others. Furthermore, a main effect of target gender emerged such that women (*M* = 24.46, *SE* = 1.31) were rated as significantly less likely to betray close others than men (*M* = 33.51, *SE* = 1.30). A main effect of viewing condition also emerged such that targets viewed with their romantic partner (*M* = 25.26, *SE* = 1.30) were rated as significantly less likely to betray close others than targets viewed alone (*M* = 32.71, *SE* = 1.30).

**Figure 1.**
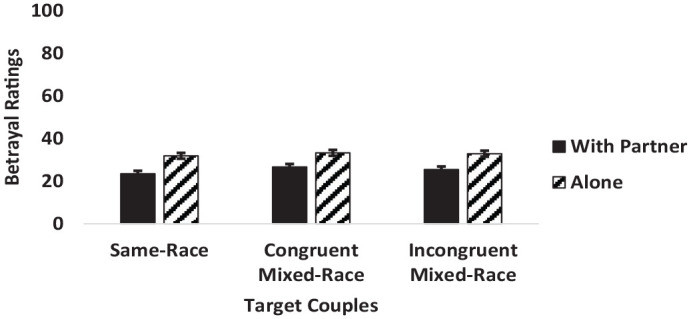
Interaction between target couples and condition on perceived betrayal ratings. *Note.*Error bars represent standard error.

A two-way interaction between couple type and viewing condition was significant (see Figure 1). Tukey’s HSD revealed that the source of the interaction emerged when target individuals were viewed with their romantic partners. In these cases, in line with H4, individuals in same-race couples were rated as less likely to betray close others than individuals in congruent mixed-race couples. No other omnibus effects were significant.

#### Conformity

A main effect of couple type emerged such that individuals in same-race couples (*M* = 61.73, *SE* = 0.96) were rated as significantly more likely to conform to societal traditions than individuals in congruent (*M* = 54.14, *SE* = 0.99) and incongruent (*M* = 52.65, *SE* = 0.99) mixed-race couples. Furthermore, individuals in congruent mixed-race couples were rated as significantly more likely to conform to societal traditions than individuals in incongruent mixed-race couples. Surprisingly, the control variable, participants’ race, was significant. In particular, White participants (*M* = 60.33, *SE* = 1.56) gave significantly higher conformity ratings than Black participants (*M* = 52.58, *SE* = 1.56), and marginally higher conformity ratings than East Asian participants (*M* = 55.62, *SE* = 1.56). There was no significant difference in conformity ratings between East Asian and Black participants.

**Figure 2.**
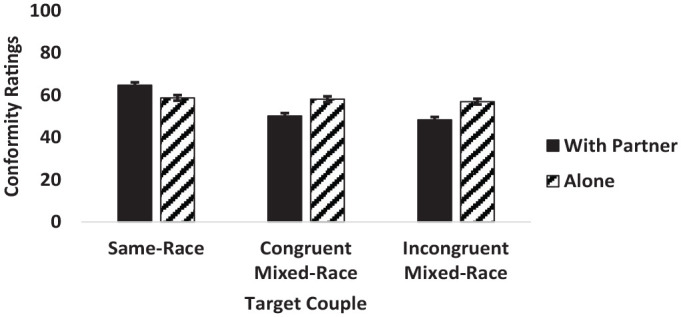
Interaction between target couples and condition on perceived conformity ratings. *Note*. Error bars represent standard error.

The two-way interaction between couple type and viewing condition was significant (see Figure 2). In line with hypotheses (H5), Tukey’s HSD revealed that individuals in same-race couples were rated as significantly more conforming, while individuals in congruent and incongruent mixed-race couples were rated as significantly less conforming when viewed with their romantic partners versus alone. Furthermore, when viewed with their romantic partners, there was a significant difference in conformity ratings between individuals in all three couple types such that individuals in same-race couples were rated as most conforming, followed by individuals in congruent mixed-race couples, followed by individuals in incongruent mixed-race couples, which were rated as least conforming. As expected, when viewed alone, there was no significant difference in perceptions of conformity between members of same-race and congruent mixed-race couples, or between congruent and incongruent mixed-race couples. However, unexpectedly, individuals in same-race couples were viewed as significantly more conforming than individuals in incongruent mixed-race couples when viewed alone.

Although the omnibus test showed that the two-way interaction between couple type and target gender, and the three-way interaction between couple type, viewing condition, and target gender achieved *p* < .05, these two effects had effect sizes that were too small to be considered meaningful, that is, *R*^2^ < .02 (Cohen, 1988). Given that we did not want to give meaning to effects that are not really there, we treated these effects as nonsignificant. No other omnibus effects emerged.

#### Prejudice

A main effect of couple type emerged such that individuals in same-race couples (*M* = 27.58, *SE* = 0.98) were rated as significantly more prejudiced than individuals in congruent (*M* = 25.86, *SE* = 0.98) and incongruent (*M* = 25.72, *SE* = 1.02) mixed-race couples. However, there was no significant difference between congruent and incongruent mixed-race couples on perceived prejudice. Furthermore, a main effect of target gender emerged such that women (*M* = 24.24, *SE* = 1.32) were rated as significantly less prejudiced than men (*M* = 28.54, *SE* = 1.28); however, we advise caution in interpreting this result because the effect size is too small to be considered meaningful (i.e., *R*^2^ < .02; Cohen, 1988). Finally, a main effect of viewing condition emerged such that targets viewed with their romantic partners (*M* = 22.46, *SE* = 1.32) were rated as significantly less prejudiced than targets viewed alone (*M* = 30.31, *SE* = 1.29).

**Figure 3.**
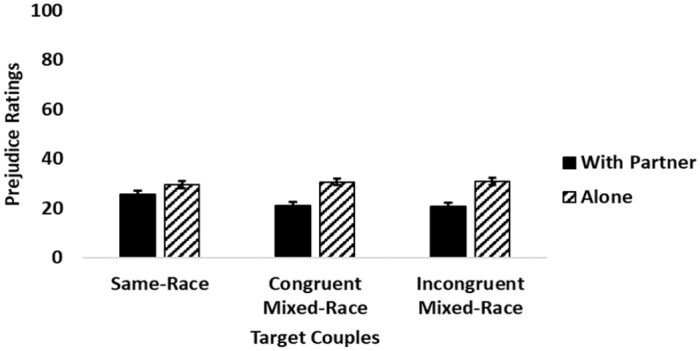
Interaction between target couples and condition on perceived prejudice ratings. *Note*. Error bars represent standard error.

In line with hypotheses (H6), the two-way interaction between couple type and viewing condition was significant (see Figure 3). Tukey’s HSD determined that individuals in congruent and incongruent mixed-race couples were rated as significantly less prejudiced when viewed with their romantic partners than alone. However, there was no significant difference in prejudice ratings for individuals in same-race couples when viewed with their romantic partners versus alone. Furthermore, individuals in same-race relationships were rated as significantly more prejudiced than individuals in congruent and incongruent mixed-race couples when viewed with their romantic partners. No other comparisons were significant.

**Figure 4.**
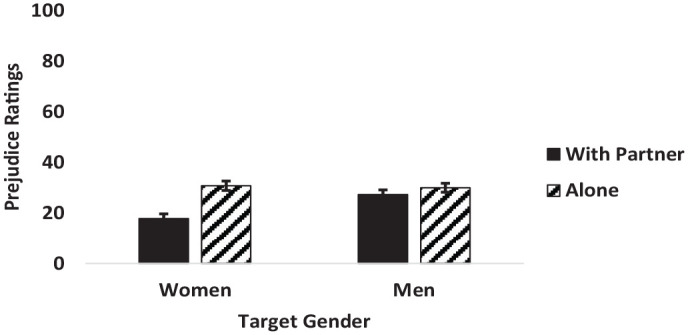
Interaction between target gender and condition on perceived prejudice ratings. *Note*. Error bars represent standard error.

A two-way interaction between target gender and viewing condition also emerged (see Figure 4). Tukey’s HSD revealed that women viewed with their romantic partners were rated as significantly less prejudiced compared to women viewed alone and to men viewed with their romantic partners. No other comparisons were significant.

In summary, across the three dependent variables measuring specific stereotypes (i.e., betrayal, conformity, and prejudice), we consistently found a main effect of couple type, which was qualified by an interaction between couple type and viewing condition. See Table 3 for a summary of the findings on specific stereotypes.

**Table 3.** Summary of findings for perceived betrayal, conformity, and prejudice.

Effect	Betrayal	Conformity	Prejudice
Couple type	*	*	*
Target gender	*		X
Viewing condition	*		*
Participants’ race		*	
Couple Type x Viewing Condition	*	*	*
Couple Type x Target Gender		X	
Target Gender x Viewing Condition			*
Three-way interaction		X	

*Note*. X Denotes a significant effect where the effect size is too small to be considered meaningful, that is, *R*^2^ < .02 (Cohen, 1988). * Denotes a significant effect where the effect size is large enough to be considered a meaningful effect, that is, *R*^2^ ⩾ .02 (Cohen, 1988).

## Discussion

In three samples, we examined people’s first impressions of individuals in same- and mixed-race couples. We found that being seen with an other-race romantic partner had little effect on general stereotypes of warmth, competence, and global morality (H1–H3). However, supporting our hypotheses, the presence of a romantic partner affected perceptions of specific stereotypic behaviors related to betrayal, conformity, and prejudice (H4–H6). Specifically, we found that individuals in mixed-race couples were perceived as more likely to betray close others, less conforming to societal traditions, and less prejudiced than individuals in same-race couples when viewed with their partners. Importantly, these effects largely did not emerge when these same individuals were seen without their romantic partner. These findings suggest that evaluations of targets on global dimensions such as warmth, competence, and global morality may not be as strongly affected by the race of their romantic partner. However, the race of the romantic partner may become an important contextual cue when considering how targets might interact with ingroup (i.e., betrayal and conformity perceptions) and outgroup members (i.e., prejudice perceptions). Given these potential implications for perceptions of intergroup relations, future research can directly examine the degree to which people believe that individuals in mixed-race relationships lose ingroup membership and gain outgroup membership.

Our study is the first to directly compare first impressions of individuals presented alone versus with their romantic partners, thus testing the impact that partner information as a contextual cue can have on perceptions of each member of a relationship. As predicted in H5, we found that individuals in mixed-race relationships were perceived to be less conforming when viewed with their partners versus alone. Also supporting our hypotheses, we found that individuals in same-race couples were viewed as most conforming, followed by individuals in stereotype-congruent mixed-race couples, followed by individuals in stereotype-incongruent mixed-race couples, which were perceived as least conforming. This could have occurred for several reasons. One potential reason why people may view incongruent mixed-race couples as especially not conforming to societal norms is that they are rarer than same-race or congruent mixed-race couples (Galinsky et al., 2013; Livingston & Brown, 2017). Furthermore, stereotype-incongruent mixed-race couples include individuals that are perceived as nonprototypical of their racial background (e.g., Schug et al., 2015), which can influence perceptions of nonconformity of these individuals in general. Indeed, individuals in our study who happened to be in incongruent mixed-race couples were perceived as less conforming than individuals in same-race couples even when viewed alone. Therefore, it could be the case that, for at least some individuals who choose to enter a stereotype-incongruent mixed-race relationship, either their own racial prototypicality and/or their mixed-race relationship could be features that perceivers use to infer a lack of conformity. Future research can explore if racial phenotypicality plays a role in perceptions of whether someone is more or less likely to follow societal norms in the context of mixed-race relationships. Furthermore, research on perceptions of individuals in groups suggests that perceptions of prototypicality of an individual may change depending on the contextual information from the group the individual is a part of (for a review, see Alt & Phillips, 2022). Therefore, future research can examine if perceptions of prototypicality (including racial phenotypicality) may differ when an individual is viewed alone versus with a partner.

In line with our prediction concerning perceived prejudice (H6), we found that individuals in mixed-race relationships were perceived as less prejudiced when viewed with their romantic partner versus alone. This finding is in line with Orta (2013), who argued that individuals in mixed-race romantic relationships should be less prejudiced because (a) partners gain knowledge about the norms, beliefs, and lifestyle of the outgroup; (b) these relationships involve feelings of love, affection, and intimacy, therefore feelings of empathy are high and feelings of anxiety or threat are low; and (c) partners may be motivated to see themselves as sharing similar characteristics and including their partners in the self. Indeed, individuals that have less ingroup bias are more likely to engage in mixed-race relationships (Levin et al., 2007). Therefore, perceivers may be accurately assessing that individuals in mixed-race relationships are less prejudiced. Future research can test whether individuals in mixed-race relationships are perceived to be less prejudiced towards different racial groups or if they are only perceived as being less prejudiced towards members of their partner’s racial group. Research can also explore whether target individuals are perceived as less prejudiced in domains other than race (e.g., religion, sexual orientation, etc.).

As for perceptions of betrayal (H4), to our surprise, members of all couples were rated as less likely to betray close others when viewed with their partners versus alone, though this decrease was greatest for same-race couples and smallest for stereotype-congruent mixed-race couples. These results suggest that being viewed with a romantic partner may have benefits in terms of perceived betrayal reduction, with same-race couples receiving the greatest benefits and congruent mixed-race couples receiving the fewest benefits. One possibility for why members of congruent mixed-race couples were perceived as most likely to betray close others may be linked to realistic conflict theory, which suggests that intergroup conflict may result from competing over limited resources (Sherif & Sherif, 1953). In a dating context, more common types of mixed-race couples, such as congruent couples (i.e., White women–Black men and East Asian women–White men), may signal to perceivers that there are fewer same-race partners available, thus making same-race partners a limited resource (Chuang et al., 2021). Indeed, qualitative interviews with Black women have found that they perceive Black men who date White women as betraying their family and community and as having weak ties with their own race, whereas Black women do not hold similar views of Black women who date White men (Childs, 2005). Recent quantitative research confirms that perceived competition over potential dating partners predicts Black women’s negative attitudes towards Black men–White women couples and Asian men’s negative attitudes towards White men–Asian women couples (Chuang et al., 2021). Therefore, if perceivers believe that individuals in congruent mixed-race couples are limiting their own dating pool of potential romantic partners, perceivers may feel threatened and betrayed by the individuals in these types of relationships.

We initially intended to compare perceptions of target individuals in each race by gender couple combination. Given that we were not able to do so due to statistical limitations, we can only draw conclusions about women and men in stereotype-congruent and stereotype-incongruent mixed-race couples, but we are not able to comment on women and men targets of any one racial group. This may be one reason why we did not find that targets’ gender interacted with type of couple in any variables. To address this, future research can compare perceptions of targets in each race by gender combination. Indeed, Stillwell and Lowery (2021) found that White women (but not Black women, White men, or Black men) are perceived as lower status when engaging in Black–White mixed-race relationships. Though comparing each race by gender combination may be desirable, there may be statistical limits as to how many couple types can be compared at once, and study designs may therefore need to limit the number of mixed-race couples included in any one comparison.

Our study design involved having different participants make ratings of targets alone versus with their partner. This allowed for the ratings of the target individuals to be unaffected by previous ratings of that same individual. However, because viewing condition was a between-groups variable, we were not able to examine how ratings of targets change between viewing individuals on their own and then viewing those same individuals again with their partners. Future research may therefore consider including viewing condition as a within-subject variable so that changes in perceptions of individuals alone versus with their partners can be examined.

One strength of our study is our inclusive samples, which consisted of approximately equal numbers of Black, East Asian, and White participants. Thus, our findings reflect the combined perceptions of individuals from various racial groups. We also found few differences between participants in various racial groups in their responding, though we acknowledge that, due to statistical limitations, we did not test whether perceptions of target individuals varied as a function of perceiver’s race (i.e., could not test moderation effects). Future research can explore the role that perceivers’ race plays in first impressions of targets in mixed-race relationships. For example, research can examine whether exposure to so many ingroup members engaging in romantic relationships with outgroup members leads perceivers to feel more positively about the outgroup members in mixed-race romantic relationships, which would be in line with extended contact theory (Zhou et al., 2019). On the other hand, as noted earlier, some recent research suggests that Black women and East Asian men may feel threatened by White women dating Black men and East Asian women dating White men, respectively, because this limits Black women’s and East Asian men’s dating options (Chuang et al., 2021). Therefore, some perceivers may respond more negatively towards individuals in these types of mixed-race relationships.

In conclusion, the present study provides the first evidence that partner race may be an important contextual cue when evaluating target individuals and makes three important contributions in this regard. First, initial impressions of individuals’ likelihood to betray close others, to conform to societal traditions, and to be prejudiced differ between individuals in mixed-race versus same-race relationships. Second, these first impressions of individuals in mixed-race relationships shift when these same people are viewed with their romantic partner versus alone. Therefore, seeing targets with an other-race romantic partner affects expectations of how individuals may function within different societal groups. These shifts in first impressions may have important downstream consequences for how perceivers themselves will interact with individuals in these relationships. For example, if someone believes that a target individual is likely to betray close others, they may not trust the individual, may distance themselves from the individual, and may be less friendly towards the individual, which has implications for behavior in a variety of contexts such as friendships, workplaces, and courtrooms. Third, we uncovered unique stereotypes that are applied to individuals depending on the type of mixed-race relationship they are in. In particular, individuals in stereotype-congruent mixed-race relationships were perceived as most likely to betray close others, while individuals in stereotype-incongruent mixed-race relationships were perceived as least conforming. Importantly, members of congruent and incongruent mixed-race couples were rated as similarly unprejudiced. Overall, the present study deepens our knowledge of the unique forms of stigma experienced by individuals in mixed-race relationships while also broadening our understanding of person perception, intergroup functioning, and intersectionality.

## Supplemental Material

sj-docx-1-gpi-10.1177_13684302241233505 – Supplemental material for Perceptions of women and men in mixed-race heterosexual relationships Supplemental material, sj-docx-1-gpi-10.1177_13684302241233505 for Perceptions of women and men in mixed-race heterosexual relationships by Maria Iankilevitch and Alison L. Chasteen in Group Processes & Intergroup Relations

## Notes

1. The analyses for all samples were conducted at about the same time and were part of an evolving learning process. Therefore, the analytic plan changed after the initial preregistration of Samples 1a and 1c because models were not converging, which is likely due to complex model structures. We had not initially anticipated model nonconvergence as a possibility and needed to then take steps to achieve model convergence. As a first step, we simplified the models to control for participant race (see Endnote 2). We then updated our preregistration of Samples 1a and 1c using this simplified model structure. This did not achieve model convergence. To address model convergence, we further simplified the model by grouping the seven gender by race couple types into same-race, stereotype-congruent mixed-race, and stereotype-incongruent mixed-race couples based on existing intersectionality literature. We also trimmed the random effects as described in the supplementary material. Therefore, we once again updated our preregistration of Samples 1a and 1c. All preregistrations were made prior to conducting the relevant planned analyses.

2. We controlled for participant race because we encountered unanticipated statistical limitations whereby the statistical models were not converging, suggesting that we needed to simplify our analytical approach. Given that other variables included in the study pertained most closely to the main research question, we decided to control for participant race rather than include it as an additional independent variable. We note, however, that a strength of our samples is that they are more inclusive than has typically been found in past person perception research.

3. See data and analysis codes for all samples on Open Science Framework: https://osf.io/mchgy/.
