# Supplementary material for: Perceptions of women and men in mixed-race heterosexual relationships
Source: Group Process Intergroup Relat. 2024 Mar 16;27(8):1757–72. doi: 10.1177/13684302241233505 (PMC11624103; doi:10.1177/13684302241233505)
Supplement: sj-docx-1-gpi-10.1177_13684302241233505 – Supplemental material for Perceptions of women and men in mixed-race heterosexual relationships [file sj-docx-1-gpi-10.1177_13684302241233505.docx]

**Perceptions of Individuals in Mixed-Race Relationships**

**Supplementary Materials**

**Table of Contents**

| Details about Trimming for Each Variable | | p. 2 |
| --- | --- | --- |
| Results of General Stereotypes | | p. 3 |
|  | Warmth | p. 4 |
|  | Competence | p. 4 |
|  | Global Morality | p. 7 |
| Post-Hoc Tables of Specific Stereotypes | | p. 9 |

**Details about Trimming for Each Variable**

The models for all dependent variables did not converge at first, therefore, we employed trimming. In particular, where needed, we trimmed the random effects in line with Bates et al. (2015a) to achieve convergence. The steps taken for each dependent variable are described below.

To achieve convergence for perceived *warmth*, we first eliminated the correlation parameters. Next, we dropped the random effect of condition which had a variance component close to zero, and then we dropped the random effect of couple type which also had a variance component close to zero. This resulted in model convergence.

For perceived *competence* and perceived *global morality*, we eliminated the correlation parameters, which resulted in model convergence for both variables. Therefore, no further trimming was needed.

To achieve convergence for perceived likelihood to *betray* close others, perceived *conformity*, and perceived *prejudice*, we first eliminated the correlation parameters of the random effects. We then dropped the random effect of condition, which had a variance component close to zero. Next, we dropped the random effect of target sex, which resulted in model convergence for perceived likelihood to *betray* and perceived *prejudice* variables. For *conformity*, we additionally dropped the random effect of couple type, which then resulted in model convergence.

**Results of General Stereotypes**

See Table S1 for omnibus statistics for Warmth, Competence, and Global Morality and Table S2 for a summary of the findings.

**Table S1**

*Omnibus Statistics for Warmth, Competence, and Global Morality*

|  | Effect | *F*(*df*) | *p* | *R^2^* |
| --- | --- | --- | --- | --- |
| Warmth | |  |  |  |
|  | Participants’ Race (Control) | .65(2, 457) | .525 | < .01 |
|  | Couple Type | 1.10(2, 137) | .337 | .02 |
|  | Target Gender | 6.70(1, 524) | .010 | .01 |
|  | Viewing Condition | 3.03(1, 457) | .083 | < .01 |
|  | Couple Type x Viewing Condition | 2.11(2, 64054) | .121 | < .01 |
|  | Couple Type x Target Gender | .37(2, 137) | .690 | < .01 |
|  | Target Gender x Viewing Condition | 2.45(1, 457) | .118 | < .01 |
|  | Three-Way Interaction | 9.97(2, 64054) | < .001 | < .01 |
| Competence | |  |  |  |
|  | Participants’ Race (Control) | 2.52(2, 457) | .082 | .01 |
|  | Couple Type | 5.25(2, 158) | .006 | .06 |
|  | Target Gender | 13.05(1, 482) | < .001 | .03 |
|  | Viewing Condition | .79(1, 459) | .373 | < .01 |
|  | Couple Type x Viewing Condition | .78(2, 256) | .461 | < .01 |
|  | Couple Type x Target Gender | .20(2, 181) | .816 | < .01 |
|  | Target Gender x Viewing Condition | 2.45(2, 457) | .118 | < .01 |
|  | Three-Way Interaction | 4.39(2, 478) | .013 | .02 |
| Global Morality | |  |  |  |
|  | Participants’ Race (Control) | .54(2, 396) | .584 | < .01 |
|  | Couple Type | 10.48(2, 248) | < .001 | .08 |
|  | Target Gender | 12.65(1, 414) | < .001 | .03 |
|  | Viewing Condition | 20.07(1, 398) | < .001 | .05 |
|  | Couple Type x Viewing Condition | 2.63(2, 328) | .073 | .02 |
|  | Couple Type x Target Gender | .50(2, 263) | .609 | < .01 |
|  | Target Gender x Viewing Condition | < .01(1, 396) | .976 | < .01 |
|  | Three-Way Interaction | 1.34(2, 398) | .263 | < .01 |

*Note*. *F* = *F*-ratio, *df* = degrees of freedom, *p* = probability value.

**Table S2**

*Summary of Findings for Warmth, Competence, and Global Morality*

| Effect | Warmth | Competence | Global Morality |
| --- | --- | --- | --- |
| Couple Type |  | * | * |
| Target Gender | X | * | * |
| Viewing Condition |  |  | * |
| Participants’ Race |  |  |  |
| Couple Type x Viewing Condition |  |  |  |
| Couple Type x Target Gender |  |  |  |
| Target Gender x Viewing Condition |  |  |  |
| Three-Way Interaction | X | * |  |

*Note*. X denotes a significant effect where the effect size is too small to be considered meaningful. * denotes a significant effect where the effect size is large enough to be considered a meaningful effect.

**Warmth**

The omnibus test showed that the main effect of target gender and the three-way interaction between couple type, condition, and target sex achieved *p* < .05. However, these effects had an effect size that was too small to be considered meaningful, that is *R*^2^ < .02 (Cohen, 1988). Given that we did not want to give meaning to effects that are not really there, we treated these effects as non-significant. No other omnibus effects emerged.

**Competence**

A significant main effect of couple type emerged. As predicted, Tukey’s HSD determined that individuals in same-race couples (*M* = 60.79, *SE* = .86) were rated as marginally more competent than individuals in congruent same-race couples (*M* = 59.63, *SE* = .89) and significantly more competent than individuals in incongruent mixed-race couples (*M* = 59.12, *SE* = .89). However, there was no significant difference between congruent and incongruent mixed-race couples on perceived competence (see Table S3). Furthermore, a main effect of target gender emerged, such that women (*M* = 62.75, *SE* = 1.14) were rated as significantly more competent than men (*M* = 56.94, *SE* = 1.16).

**Table S3**

*Post-Hoc Analyses of Main Effect of Couple Type for Perceived Competence*

| Groups Compared | *b*(*SE*) | *Z* | OR [95% CI] |
| --- | --- | --- | --- |
| same vs. congruent | 1.15(.53) | 2.18^t^ | 3.17 [1.12, 8.96] |
| same vs. incongruent | 1.67(.55) | 3.06^**^ | 5.32 [1.82, 15.5] |
| congruent vs. incongruent | .52(.59) | .87 | 1.68 [.52, 5.37] |

*Note*. Same = target individuals in same-race couples; congruent = target individuals in congruent mixed-race couples, incongruent = target individuals in incongruent mixed-race couples, *b* = unstandardized beta, *SE* = standard error, *z* = Z score, OR = Odds Ratio, 95% CI = 95% confidence interval, ^t^ = .05 < *p* < .10, ** *p* < .01.

A significant three-way interaction emerged between couple type, viewing condition, and target sex. However, the results of Tukey’s HSD were largely non-significant (see Table S4 for post hoc statistics and Figure S1 for pattern of results). In particular, men and women in all couples were rated as similarly competent when viewed with their romantic partners versus alone. Furthermore, there was no significant difference in competence ratings for men and women comparing different couple types when viewed with their romantic partners. There was also no significant difference in competence ratings for women that are part of different couple types when viewed alone. Similarly, there was no significant difference in competence ratings between men in same-race versus congruent mixed-race couples and congruent versus incongruent mixed-race couples when viewed alone. The only significant difference found was that men that are part of same-race couples were rated as significantly more competent than men that are part of incongruent mixed-race couples when viewed alone.

No other omnibus effects emerged.

**Table S4**

*Post Hoc Results for Three-Way Interaction for Perceived Competence*

| Female Targets | | | |
| --- | --- | --- | --- |
|  | *b*(*SE*) | *z* | OR [95% CI] |
| Same-Race Couples: With Partner vs. Alone | -.70(2.25) | -.31 | 2.01  [.02, 166.65] |
| Congruent Mixed-Race Couples: With Partner vs. Alone | -1.09(2.26) | -.48 | 2.99  [.04, 251.34] |
| Incongruent Mixed-Race Couples: With Partner vs. Alone | -1.48(2.27) | -.65 | 4.39  [.05, 376.80] |
| With Partner: Same-Race Couples vs. Congruent Mixed-Race Couples | 1.13(.69) | 1.63 | 3.08  [.79, 11.99] |
| With Partner: Same-Race Couples vs. Incongruent Mixed-Race Couples | 2.04(.74) | 2.76 | 7.67  [1.81, 32.59] |
| With Partner: Congruent Mixed-Race Couples vs. Incongruent Mixed-Race Couples | .91(.80) | 1.14 | 2.49  [.52, 11.86] |
| Alone: Same-Race Couples vs. Congruent Mixed-Race Couples | .73(.70) | 1.05 | 2.08  [.53, 8.18] |
| Alone: Same-Race Couples vs. Incongruent Mixed-Race Couples | 1.26(.75) | 1.68 | 3.52  [.81, 15.23] |
| Alone: Congruent Mixed-Race Couples vs. Incongruent Mixed-Race Couples | .53(.81) | .65 | 1.69  [.35, 8.22] |
| Male Targets | | | |
|  | *b*(*SE*) | *z* | OR [95% CI] |
| Same-Race Couples: With Partner vs. Alone | 3.22(2.29) | 1.41 | 25.11  [.28, 2247.48] |
| Congruent Mixed-Race Couples: With Partner vs. Alone | 3.50(2.30) | 1.52 | 33.16  [.36, 3017.08] |
| Incongruent Mixed-Race Couples: With Partner vs. Alone | 5.04(2.31) | 2.18 | 154.16  [1.66, 14306.57] |
| With Partner: Same-Race Couples vs. Congruent Mixed-Race Couples | 1.24(.70) | 1.77 | 3.46  [.87, 13.68] |
| With Partner: Same-Race Couples vs. Incongruent Mixed-Race Couples | .79(.75) | 1.05 | 2.20  [.50, 9.59] |
| With Partner: Congruent Mixed-Race Couples vs. Incongruent Mixed-Race Couples | -.45(.81) | -.56 | 1.57  [.32, 7.69] |
| Alone: Same-Race Couples vs. Congruent Mixed-Race Couples | 1.52(.70) | 2.18 | 4.56  [1.17, 17.84] |
| Alone: Same-Race Couples vs. Incongruent Mixed-Race Couples | 2.60(.74) | 3.51^*^ | 13.51  [1.96, 92.88] |
| Alone: Congruent Mixed-Race Couples vs. Incongruent Mixed-Race Couples | 1.08(.80) | 1.35 | 2.96  [.62, 14.22] |

*Note*. *b* = unstandardized beta, *SE* = standard error, *z* = Z score, OR = Odds Ratio, 95% CI = 95% confidence interval, ^*^ = *p* < .05.

**Figure S1**

*Interaction between Target Couples, Condition, and Target Gender on Perceived Competence*

*Ratings*

*
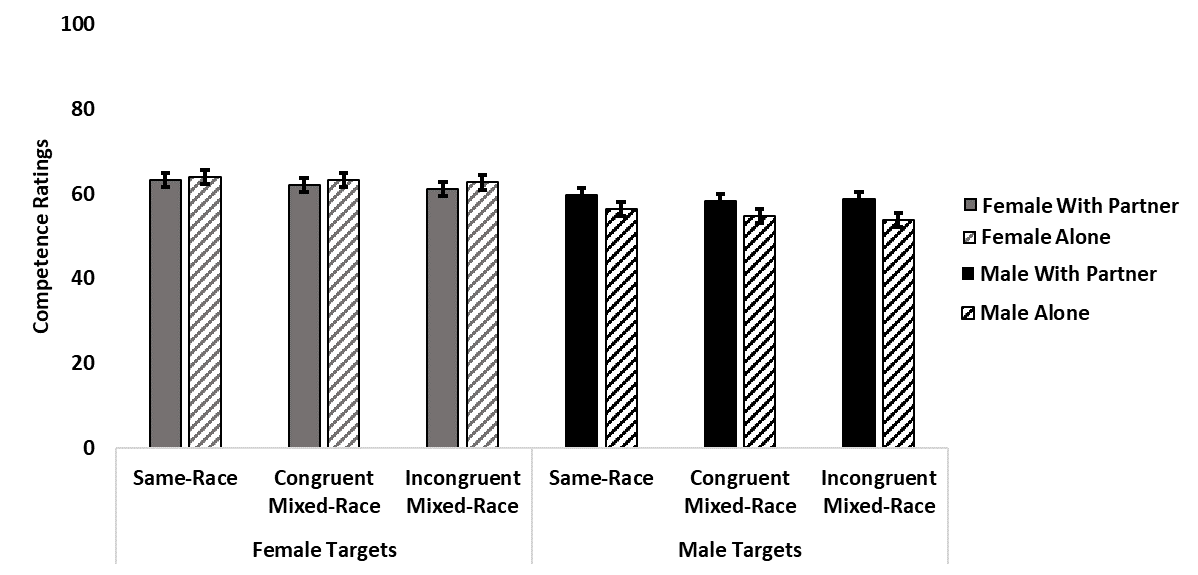
*

*Note*. Error bars represent standard error.

**Global Morality**

A main effect of couple type emerged. As predicted, Tukey’s HSD determined that individuals in same-race couples (*M* = 64.91, *SE* = .92) were rated as more moral than individuals in congruent (*M* = 63.17, *SE* = .93) and in incongruent (*M* = 62.96, *SE* = .94) mixed-race couples. However, in contrast to the hypotheses, there was no significant difference between congruent mixed-race and incongruent mixed-race couples on perceived morality (see Table S5). Furthermore, a main effect of target gender emerged, such that women (*M* = 66.83, *SE* = 1.25) were rated as more moral than men (*M* = 60.53, *SE* = 1.26). Finally, a main effect of viewing condition emerged, such that targets viewed with their romantic partners (*M* = 67.60, *SE* = 1.24) were rated as more moral than targets viewed alone (*M* = 59.75, *SE* = 1.25). No other effects emerged.

**Table S5**

*Post-Hoc Analyses of Main Effect of Couple Type for Perceived Global Morality*

| Groups Compared | *b*(*SE*) | *Z* | OR [95% CI] |
| --- | --- | --- | --- |
| same vs. congruent | 1.74(.43) | 4.06^***^ | 5.69 [2.46, 13.20] |
| same vs. incongruent | 1.95(.52) | 3.77^***^ | 7.04 [2.55, 19.04] |
| congruent vs. incongruent | .21(.49) | .43 | 1.24 [.47, 3.26] |

*Note*. Same = target individuals in same-race couples; congruent = target individuals in congruent mixed-race couples, incongruent = target individuals in incongruent mixed-race couples, *b* = unstandardized beta, *SE* = standard error, *z* = Z score, OR = Odds Ratio, 95% CI = 95% confidence interval, *** = *p* < .001.

**Post-Hoc Tables of Specific Stereotypes**

See Tables S6-S10 for statistics of post-hoc analyses for perceived betrayal, conformity, and prejudice.

**Table S6**

*Post-Hoc Analyses of Significant Main Effects*

| Variable | Groups Compared | *b*(*SE*) | *Z* | OR [95% CI] |
| --- | --- | --- | --- | --- |
| Betrayal |  |  |  |  |
|  | same vs. congruent | -2.34(.60) | -3.91^***^ | 10.34 [3.21, 33.34] |
|  | same vs. incongruent | -1.54(.69) | -2.25^t^ | 4.68 [1.22, 17.98] |
|  | congruent vs. incongruent | .79(.68) | 1.16 | 2.21 [.58, 8.41] |
| Conformity |  |  |  |  |
|  | same vs. congruent | 7.59(.55) | 13.93^***^ | 1978.31  [679.80, 5757.16] |
|  | same vs. incongruent | 9.09(.55) | 16.67^***^ | 8866.19  [3046.66, 25801.81] |
|  | congruent vs. incongruent | 1.50(.60) | 2.51^*^ | 4.48  [1.39, 14.44] |
|  | White vs. East Asian Participants | 4.71(2.19) | 2.15^t^ | 111.05  [1.52, 8122.56] |
|  | White vs. Black Participants | 7.75(2.19) | 3.54^**^ | 2321.57  [31.74, 169804.00] |
|  | East Asian vs. Black Participants | 3.04(2.19) | 1.39 | 20.91  [.29, 1529.05] |
| Prejudice |  |  |  |  |
|  | same vs. congruent | 1.72(.53) | 3.23^**^ | 5.57 [1.97, 15.76] |
|  | same vs. incongruent | 1.86(.69) | 2.70^*^ | 6.42 [1.66, 24.77] |
|  | congruent vs. incongruent | .14(.63) | .23 | 1.15 [.33, 3.95] |

*Note*. Same = target individuals in same-race couples; congruent = target individuals in congruent mixed-race couples, incongruent = target individuals in incongruent mixed-race couples, *b* = unstandardized beta, *SE* = standard error, *z* = Z score, OR = Odds Ratio, 95% CI = 95% confidence interval, ^t^ = .05 < *p* < .10, * *p* < .05, ** *p* < .01, *** = *p* < .001.

**Table S7**

*Perceived Betrayal: Post Hoc Results for Interaction between Couple Type and Condition*

|  | *b*(*SE*) | *z* | OR [95% CI] |
| --- | --- | --- | --- |
| Same-Race Couples: With Partner vs. Alone | -8.35(1.87) | -4.47^***^ | 4217.49  [108.82, 163451.50] |
| Congruent Mixed-Race Couples: With Partner vs. Alone | -6.58(1.85) | -3.56^**^ | 719.63  [19.27, 26879.06] |
| Incongruent Mixed-Race Couples: With Partner vs. Alone | -7.42(1.89) | -3.93^**^ | 1661.03  [41.02, 67252.92] |
| With Partner: Same-Race Couples vs. Congruent Mixed-Race Couples | -3.22(.66) | -4.87^***^ | 25.04  [6.85, 91.49] |
| With Partner: Same-Race Couples vs. Incongruent Mixed-Race Couples | -2.01(.82) | -2.46 | 7.45  [1.50, 36.98] |
| With Partner: Congruent Mixed-Race Couples vs. Incongruent Mixed-Race Couples | 1.21(.77) | 1.57 | 3.36  [.74, 15.27] |
| Alone: Same-Race Couples vs. Congruent Mixed-Race Couples | -1.45(.66) | -2.20 | 4.27  [1.17, 15.61] |
| Alone: Same-Race Couples vs. Incongruent Mixed-Race Couples | -1.08(.82) | -1.32 | 2.94  [.59, 14.56] |
| Alone: Congruent Mixed-Race Couples vs. Incongruent Mixed-Race Couples | .38(.77) | .49 | 1.46  [.32, 6.62] |

*Note*. *b* = unstandardized beta, *SE* = standard error, *z* = Z score, OR = Odds Ratio, 95% CI = 95% confidence interval, ^**^ = *p* < .01, ^***^ = *p* < .001.

**Table S8**

*Perceived Conformity: Post Hoc Results for Interaction between Couple Type and Condition*

|  | *b*(*SE*) | *z* | OR [95% CI] |
| --- | --- | --- | --- |
| Same-Race Couples: With Partner vs. Alone | 6.04(1.80) | 3.36^*^ | 418.22  [12.38, 14131.48] |
| Congruent Mixed-Race Couples: With Partner vs. Alone | -7.93(1.80) | -4.40^***^ | 2765.56  [80.73, 94738.90] |
| Incongruent Mixed-Race Couples: With Partner vs. Alone | -8.59(1.80) | -4.77^***^ | 5399.17  [157.61, 184957.30] |
| With Partner: Same-Race Couples vs. Congruent Mixed-Race Couples | 14.57(.57) | 25.41^***^ | 2130783.00  [6550684.00, 693093.40] |
| With Partner: Same-Race Couples vs. Incongruent Mixed-Race Couples | 16.40(.57) | 28.61^***^ | 13296348.00  [40877077.00, 4324988.00] |
| With Partner: Congruent Mixed-Race Couples vs. Incongruent Mixed-Race Couples | 1.83(.63) | 2.92^*^ | 6.24  [1.82, 21.37] |
| Alone: Same-Race Couples vs. Congruent Mixed-Race Couples | .61(.57) | 1.07 | 1.84  [.60, 5.66] |
| Alone: Same-Race Couples vs. Incongruent Mixed-Race Couples | 1.77(.57) | 3.09^*^ | 5.89  [1.92, 18.10] |
| Alone: Congruent Mixed-Race Couples vs. Incongruent Mixed-Race Couples | 1.16(.63) | 1.85 | 3.20  [.93, 10.94] |

*Note*. *b* = unstandardized beta, *SE* = standard error, *z* = Z score, OR = Odds Ratio, 95% CI = 95% confidence interval, ^*^ = *p* < .05, ^***^ = *p* < .001.

**Table S9**

*Perceived Prejudice: Post Hoc Results for Interaction between Couple Type and Condition*

|  | *b*(*SE*) | *z* | OR [95% CI] |
| --- | --- | --- | --- |
| Same-Race Couples: With Partner vs. Alone | -4.00(1.90) | -2.10 | 54.43  [1.32, 2250.80] |
| Congruent Mixed-Race Couples: With Partner vs. Alone | -9.42(1.86) | -5.06^***^ | 12344.92  [320.38, 475670.2] |
| Incongruent Mixed-Race Couples: With Partner vs. Alone | -10.12(1.93) | -5.24^***^ | 24809.95  [564.65, 1090122] |
| With Partner: Same-Race Couples vs. Congruent Mixed-Race Couples | 4.43(.63) | 6.99^***^ | 83.85  [24.25, 289.94] |
| With Partner: Same-Race Couples vs. Incongruent Mixed-Race Couples | 4.92(.89) | 5.51^***^ | 137.00  [23.85, 787.07] |
| With Partner: Congruent Mixed-Race Couples vs. Incongruent Mixed-Race Couples | .49(.77) | .63 | 1.63  [.36, 7.43] |
| Alone: Same-Race Couples vs. Congruent Mixed-Race Couples | -1.00(.62) | -1.59 | 2.70  [.80, 9.19] |
| Alone: Same-Race Couples vs. Incongruent Mixed-Race Couples | -1.20(.88) | -1.37 | 3.33  [.60, 18.49] |
| Alone: Congruent Mixed-Race Couples vs. Incongruent Mixed-Race Couples | -.21(.76) | -.27 | 1.23  [.28, 5.46] |

*Note*. *b* = unstandardized beta, *SE* = standard error, *z* = Z score, OR = Odds Ratio, 95% CI = 95% confidence interval, ^***^ = *p* < .001.

**Table S10**

*Perceived Prejudice: Post Hoc Results for Interaction between Target Gender and Condition*

|  | *b*(*SE*) | *z* | OR [95% CI] |
| --- | --- | --- | --- |
| Women Targets: With Partner vs. Alone | -12.99(2.62) | -4.95^***^ | 438011.3  [2578.08, 74417374] |
| Men Targets: With Partner vs. Alone | -2.70(2.54) | -1.07 | 14.91  [.10, 2165.49] |
| With Partner: Women vs. Men Targets | -9.44(2.61) | -3.62^**^ | 12594.3  [75.60, 2098220] |
| Alone: Women vs. Men Targets | .85(2.55) | .33 | 2.33  [.02, 345.16] |

*Note*. *b* = unstandardized beta, *SE* = standard error, *z* = Z score, OR = Odds Ratio, 95% CI = 95% confidence interval, ^**^ = *p* < .01, ^***^ = *p* < .001.
